# Supplementary material for: Myelin plasticity in the ventral tegmental area is required for opioid reward
Source: Nature. Author manuscript; Available in PMC 2024 Aug 9. (PMC11186775; doi:10.1038/s41586-024-07525-7)
Supplement: SI guide [file NIHMS2002924-supplement-SI_guide.docx]

**Myelin plasticity in ventral tegmental area is required for opioid reward**

Belgin Yalçın^1^, Matthew B. Pomrenze^2^, Karen Malacon^1^, Richard Drexler^1^, Abigail E. Rogers^1^, Kiarash Shamardani^1^, Isabelle J. Chau^1^, Kathryn Taylor^1^, Lijun Ni^1^, Daniel Contreras-Esquivel^1^, Robert C. Malenka^2^, Michelle Monje^1, 2, 3*^

1 Department of Neurology and Neurological Sciences, Stanford University, Stanford CA,

2 Nancy Pritzker Laboratory, Department of Psychiatry and Behavioral Sciences, Stanford University, Stanford CA,

1. Howard Hughes Medical Institute, Stanford, CA

*Corresponding author

**Supplementary Information:**

Source Data – excel file with all raw data, organized by figure panel.
