# Supplementary material for: Myelin plasticity in the ventral tegmental area is required for opioid reward
Source: Nature. 2024 Jun 5;630(8017):677–85. doi: 10.1038/s41586-024-07525-7 (PMC11186775; doi:10.1038/s41586-024-07525-7)
Supplement: Supplementary file 1 [file 41586_2024_7525_MOESM1_ESM.pdf]

nature portfolio

Corresponding author(s): Michelle Monje

Last updated by author(s): Mar 4, 2024

Reporting Summary

Nature Portfolio wishes to improve the reproducibility of the work that we publish. This form provides structure for consistency and transparency in reporting. For further information on Nature Portfolio policies, see our Editorial Policies and the Editorial Policy Checklist.

Statistics

For all statistical analyses, confirm that the following items are present in the figure legend, table legend, main text, or Methods section.

n/a	Confirmed	
☐	☒	The exact sample size (*n*) for each experimental group/condition, given as a discrete number and unit of measurement
☐	☒	A statement on whether measurements were taken from distinct samples or whether the same sample was measured repeatedly
☐	☒	The statistical test(s) used AND whether they are one- or two-sided<br>*Only common tests should be described solely by name; describe more complex techniques in the Methods section.*
☐	☒	A description of all covariates tested
☐	☒	A description of any assumptions or corrections, such as tests of normality and adjustment for multiple comparisons
☐	☒	A full description of the statistical parameters including central tendency (e.g. means) or other basic estimates (e.g. regression coefficient) AND variation (e.g. standard deviation) or associated estimates of uncertainty (e.g. confidence intervals)
☒	☐	For null hypothesis testing, the test statistic (e.g. *F*, *t*, *r*) with confidence intervals, effect sizes, degrees of freedom and *P* value noted<br>*Give P values as exact values whenever suitable.*
☒	☐	For Bayesian analysis, information on the choice of priors and Markov chain Monte Carlo settings
☒	☐	For hierarchical and complex designs, identification of the appropriate level for tests and full reporting of outcomes
☒	☐	Estimates of effect sizes (e.g. Cohen's *d*, Pearson's *r*), indicating how they were calculated

Our web collection on statistics for biologists contains articles on many of the points above.

Software and code

Policy information about availability of computer code

Data collection	All behavioral data were recoded as videos.
Data analysis	Behavioral experiments were analyzed using Ethovision XT software (Noldus) in an automated and condition-blinded manner. Fiji was used for confocal and electron microscopy analysis. MBF Stereoinvestigator is used for analysis of fluorescence stereomicropscope images. All statistical analysis were performed using Graphad Prism Software. Fiber photometry signal processing was performed in MATLAB (MathWorks).

For manuscripts utilizing custom algorithms or software that are central to the research but not yet described in published literature, software must be made available to editors and reviewers. We strongly encourage code deposition in a community repository (e.g. GitHub). See the Nature Portfolio guidelines for submitting code & software for further information.

Data

Policy information about availability of data

All manuscripts must include a data availability statement. This statement should provide the following information, where applicable:

- Accession codes, unique identifiers, or web links for publicly available datasets
- A description of any restrictions on data availability
- For clinical datasets or third party data, please ensure that the statement adheres to our policy

All data are available in the main text or the supplementary materials. Sourced data will be available with the final version of the manuscript.

Human research participants

Policy information about studies involving human research participants and Sex and Gender in Research.

Reporting on sex and gender	*Use the terms sex (biological attribute) and gender (shaped by social and cultural circumstances) carefully in order to avoid confusing both terms. Indicate if findings apply to only one sex or gender; describe whether sex and gender were considered in study design whether sex and/or gender was determined based on self-reporting or assigned and methods used. Provide in the source data disaggregated sex and gender data where this information has been collected, and consent has been obtained for sharing of individual-level data; provide overall numbers in this Reporting Summary. Please state if this information has not been collected. Report sex- and gender-based analyses where performed, justify reasons for lack of sex- and gender-based analysis.*
Population characteristics	*Describe the covariate-relevant population characteristics of the human research participants (e.g. age, genotypic information, past and current diagnosis and treatment categories). If you filled out the behavioural & social sciences study design questions and have nothing to add here, write "See above."*
Recruitment	*Describe how participants were recruited. Outline any potential self-selection bias or other biases that may be present and how these are likely to impact results.*
Ethics oversight	*Identify the organization(s) that approved the study protocol.*

Note that full information on the approval of the study protocol must also be provided in the manuscript.

Field-specific reporting

Please select the one below that is the best fit for your research. If you are not sure, read the appropriate sections before making your selection.

☒ Life sciences ☐ Behavioural & social sciences ☐ Ecological, evolutionary & environmental sciences

For a reference copy of the document with all sections, see nature.com/documents/nr-reporting-summary-flat.pdf

Life sciences study design

All studies must disclose on these points even when the disclosure is negative.

Sample size	. Sample sizes were guided by power calculation and based on previous publications (Gibson et al 2014, Science) Sample sizes of 3–20 animals were sufficient to determine significance both in histological analysis and behaviour tests.
Data exclusions	For CPP tests, mice that show strong preference (>75%) to either chamber during PreTest was excluded from further analysis. For novel object recognition, animals that explored the objects less than 20 seconds were excluded from the analysis. Brain tissue that is damaged during perfusion or tissue processing, or with inadequate viral labeling were excluded from histological analysis.
Replication	Every experiment were repeated multiple times to replicate in independent cohorts of animals, and all attempts at replication was successful. Reported findings were reproduced across animals in histological, and behavioral experiments. Total number of animals were reported in each figure, and group means were used for statistical analysis as indicated in the figure legends, and methods section.
Randomization	Animals were randomized by cage prior to surgeries. For example, mice were randomly assigned to YFP or ChR2 groups, or wild type animals were randomized for receiving saline, morphine, or cocaine injections.
Blinding	All behavioral and histological analysis were conducted blind to genotype, manipulation or to drug treatment.

Reporting for specific materials, systems and methods

We require information from authors about some types of materials, experimental systems and methods used in many studies. Here, indicate whether each material, system or method listed is relevant to your study. If you are not sure if a list item applies to your research, read the appropriate section before selecting a response.

Materials & experimental systems

n/a	Involved in the study
☐	☒ Antibodies
☒	☐ Eukaryotic cell lines
☒	☐ Palaeontology and archaeology
☐	☒ Animals and other organisms
☒	☐ Clinical data
☒	☐ Dual use research of concern

Methods

n/a	Involved in the study
☒	☐ ChIP-seq
☒	☐ Flow cytometry
☒	☐ MRI-based neuroimaging

Antibodies

Antibodies used

Primary antibodies: Goat anti- Pdgfra (1:500; R&D Systems, AF1062), rabbit anti-Olig2 (1:500; Abcam, 7349), rabbit anti-ASPA (1:250; EMD Millipore, ABN1698), rat anti-MBP (1:200; Abcam ab7349), rabbit anti-tyrosine hydroxylase (1:500; Millipore Sigma, AB152), mouse anti-tyrosine hydroxylase (1:250; Novus Biologicals, MAB7566), chicken anti-GFP (1:1000; Aves Labs, GFP-1020), chicken anti-mCherry (1:1000; ab205402), or rabbit anti-cfos (1:500; Santa Cruz Biotechnology, sc-52).
secondary antibodies: used at 1:500 concentration including Alexa 488 anti-rabbit (Jackson ImmunoResearch; 711-545-152), Alexa 488 anti-mouse (Jackson ImmunoResearch; 715-545-150), Alexa 488 anti-chicken (Jackson ImmunoResearch; 703-545-155), Alexa 594 anti-chicken (Jackson ImmunoResearch; 703-585-155), Alexa 647 donkey anti-goat (Jackson ImmunoResearch; 705-605-147), Alexa 647 anti-rat (Jackson ImmunoResearch; 712-605-150), Alexa 647 anti-rabbit (Jackson ImmunoResearch; 711-605-152), Alexa 647 donkey anti-goat (Jackson ImmunoResearch; 705-605-147). All used at 1:500

Validation

Immunostaining results were compared with published data for known distribution of labeled structures.

Animals and other research organisms

Policy information about studies involving animals; ARRIVE guidelines recommended for reporting animal research, and Sex and Gender in Research

Laboratory animals

Hemizygous Dat-Cre (The Jackson Laboratory, 006660) mice were bred with C57BL/6J (The Jackson Laboratory, 000664). Homozygous for Vgat-Cre (The Jackson Laboratory, 028862) mice bred with C57BL/6J (The Jackson Laboratory, 000664). For conditional deletion of Myrf, hemizygous Pdgfra-CreERTM (The Jackson Laboratory, 018280) mice were bred with homozygous Myrflox/lox (The Jackson Laboratory, 010607). For conditional deletion of TrkB, hemizygous Pdgfra-CreERTM (The Jackson Laboratory, 018280) mice were bred with homozygous TrkBlox/lox (MMRC, 033048-UCD). For conditional deletion of Oprk, hemizygous Pdgfra-CreERTM (The Jackson Laboratory, 018280) mice were bred with homozygous Oprk1lox/lox (The Jackson Laboratory, 030076). For oligodendrocyte specific expression of mGFP, homozygous mT/mGlox/lox (The Jackson Laboratory, 007676) animals were crossed with Plp1-CreERT (The Jackson Laboratory, 005975). For testing morphine effects on oligodendroglial cells, wild type C57BL/6J (The Jackson Laboratory, 000664) mice were used. All animals for housed in a 12-hour light/dark cycle with unrestricted access to food and water. Behavioral experiments were performed during the same circadian period (7:00-19:00).

Wild animals

No wild animals were used.

Reporting on sex

Both male and female mice were used equally in all experiments.

Field-collected samples

No Samples were field-collected.

Ethics oversight

All procedures were performed in accordance with guidelines set in place by National Institutes of Health and approved by the Stanford University Institutional Care and Use Committee (IACUC).

Note that full information on the approval of the study protocol must also be provided in the manuscript.
